# Construct Validity and Test–Retest Reliability of the Al Balance Stabilometric System in Healthy Young Adults

**DOI:** 10.3390/s26216757

**Published:** 2026-10-26

**Authors:** Nilay Arman, Elçin Akyürek, Asena Yekdaneh, İrem Kurt Ulusoy, Bülent Bayraktar, Türker Şahinkaya, Ayşe Zengin Alpözgen

**Affiliations:** 1 Department of Physiotherapy and Rehabilitation, Faculty of Health Sciences, Istanbul University-Cerrahpaşa, Istanbul 34147, Türkiye; nilayarman@iuc.edu.tr; 2 Department of Physiotherapy and Rehabilitation, Institute of Graduate Studies, Istanbul University-Cerrahpaşa, Istanbul 34320, Türkiye; oskanelcin@gmail.com (E.A.); fztiremkurt@gmail.com (İ.K.U.); 3 Program of Physiotherapy (English), Vocational School of Health Services, Fenerbahçe University, Istanbul 34758, Türkiye; asenayekdaneh@gmail.com; 4 Department of Sports Medicine, Istanbul Faculty of Medicine, Istanbul University, Istanbul 34093, Türkiye; bulent.bayraktar@gmail.com (B.B.); turkerss@gmail.com (T.Ş.)

**Keywords:** postural balance, validity, reliability, stabilometry, force platform

## Abstract

**Highlights:**

**What are the main findings?**

- Al Balance scores responded to the expected sensory manipulations: sway increased when vision was removed, when the surface became compliant, and most of all when both were combined.
- Repeat testing one week apart in healthy young adults showed outcome-dependent reliability, ranging from moderate to excellent and predominantly good to excellent.

**What are the implications of the main findings?**

- Outcome-specific SEM and MDC_95_ values indicate the change needed to exceed measurement error under this protocol; they do not by themselves define clinically important change.
- Scores are device-specific: repeated assessments should use the same system, as values cannot be transferred to or from the Biodex System.

**Abstract:**

Each instrumented balance platform requires its own measurement-property evidence. We examined the test–retest reliability of the Al Balance Stabilometric System and its hypothesis-based construct validity relative to the Biodex Balance System SD. Forty healthy adults aged 18–25 years completed four Al Balance modules: four-condition sensory assessment (mCTSIB), Limits of Stability (LoS), unilateral stance (Bilateral Comparison Test), and Postural Stability Test. The matched sensory conditions, unilateral stance, and LoS were also performed on Biodex in randomized device order; Al Balance was repeated after 7 days. Validity was assessed with repeated-measures ANOVAs, repeated-measures correlations (r_rm_), within-participant profiles and standardized interaction models; reliability was assessed with intraclass correlation coefficients (ICC), standard error of measurement and minimal detectable change. Both systems captured the expected visual and surface effects, and cross-device convergence, rather than numerical agreement, was supported for the sensory conditions (r_rm_ = 0.513–0.598). However, individual-level agreement was weak: between-device correlations of participant-level sensory response magnitudes were non-significant after Holm correction, and individual asymmetry scores in unilateral stance converged weakly. Device × condition interactions indicated differing relative sensitivity. Test–retest reliability was outcome-dependent, moderate to excellent (ICC(A,1) = 0.678–0.946). In healthy young adults, Al Balance provided repeatable scores that are device-specific rather than interchangeable with Biodex.

## 1. Introduction

Balance is defined as the ability to maintain the body’s center of gravity over the base of support [1]. Although balance and postural stability are often used interchangeably, postural stability refers more specifically to the ability to maintain or recover a stable posture following disturbances or surface changes [2]. These functions rely on the integration of multiple sensory, motor, and cognitive systems [3]. During upright stance, the central nervous system continuously combines visual, vestibular, and somatosensory information and reweights these inputs when one of them becomes unavailable or unreliable, for example when the eyes are closed or the support surface is compliant [4,5]. Impairments in postural control can negatively affect daily living, independence, and quality of life. Therefore, objective and multidimensional evaluation of balance is critical in both clinical and research settings [6].

When one sensory input is impaired, as in peripheral vestibular hypofunction, postural control becomes more dependent on the remaining inputs, and instability typically increases when vision or support-surface information is also challenged. Rehabilitation of such balance disorders therefore commonly combines gaze-stability exercises, static and dynamic balance training under progressively altered visual and support-surface conditions, and gait training [7]. Planning and monitoring these interventions require measures that can quantify how standing postural control changes across sensory conditions and over time [6].

Clinical tests such as the Berg Balance Scale and Functional Reach Test are inexpensive, require little equipment, and reflect functional performance, but they are limited in precision, are subject to evaluator bias [8], and provide little information on the direction and magnitude of postural sway. Instrumented platforms complement these tests by quantifying sway under standardized conditions and by providing continuous scores suited to repeated measurement, although they require dedicated hardware and are less widely available in routine clinical settings [1,6]. The Biodex Balance System SD (BBS) is widely used in postural control assessment, and its reliability and validity have been examined for several tasks and populations, although the reported coefficients vary with protocol, outcome, and sample [9,10,11,12]. Because platforms differ in hardware, signal acquisition, and proprietary scoring algorithms, the measurement properties established for one system cannot be assumed for another [1,6].

The Al Balance Stabilometric System (Alrenas Technology Inc., Istanbul, Türkiye) is a static force platform integrated with a touchscreen computer that provides real-time data acquisition, structured patient record management, and analysis through a standard computer interface. In addition to standardized assessment modules for sensory-condition testing, limits of stability, unilateral stance, and quiet standing, it includes separate game-based training modules in which artificial intelligence generates personalized exercise plans from assessment results [13]. Because the assessment scores are intended both to describe postural control and to guide individualized training, their measurement properties are particularly important: exercise plans based on scores that do not respond to known sensory challenges, or that vary between sessions, could be misdirected. To our knowledge, these measurement properties have not yet been reported in a peer-reviewed study. The present study therefore focused on the assessment modules; the training functions were not evaluated.

Because no gold standard exists for platform-derived balance scores, and because the two systems use different hardware and proprietary scoring algorithms, numerically equivalent values were not expected. Instead, the BBS was used as an established comparator to test a priori hypotheses about convergent responses to sensory manipulation, which corresponds to hypothesis-testing construct validity rather than criterion validity [14]. We hypothesized that (1) occluding vision would worsen balance scores, (2) standing on a foam surface would worsen balance scores, (3) the greatest deterioration would occur in the eyes-closed/foam condition, and (4) participants’ changes across sensory conditions would be positively correlated between the two devices. Therefore, this study aimed to examine the test–retest reliability of the Al Balance System and to evaluate its construct validity relative to the BBS for standing postural control under altered visual and support-surface conditions and during unilateral stance; voluntary weight shifting (Limits of Stability, LoS) and quiet standing (Postural Stability Test, PST) were evaluated for reliability only.

## 2. Materials and Methods

### 2.1. Participants

Participants were recruited through convenience sampling from a community of healthy young adults living in Istanbul between June and November 2024. General announcements were made through institutional channels of physiotherapy and sports medicine departments, as well as via digital platforms and local postings to invite volunteers, who were subsequently screened for eligibility. Inclusion criteria were: (1) being between 18 and 25 years of age, (2) having a body mass index (BMI) below 24 kg/m^2^, and (3) volunteering to participate in the study. Exclusion criteria included: (1) presence of any neuromuscular or rheumatic disease, or vestibular problems that could affect balance; (2) any uncorrected visual impairment; (3) participation in exercise or physical activity that could influence trunk muscle strength or balance during the study period; (4) a history of recent orthopedic injury or surgery in the lower extremities; and (5) experiencing any acute condition between the two test sessions (within 7 days) that could affect postural stability and balance. Participants were instructed to avoid exercise, balance training, and practice of the test tasks between the two sessions, and adherence to this instruction was checked by verbal questioning at the start of the Day 7 session.

All participants were informed about the study procedures prior to participation. All assessments were conducted at the Department of Sports Medicine, Istanbul Faculty of Medicine, Istanbul University. An a priori sample-size calculation was performed using G*Power version 3.1.9.7 (Heinrich Heine University Düsseldorf, Düsseldorf, Germany). The calculation was based on the prespecified effect of sensory manipulation on the primary overall (OA) sway outcome, expressed as a standardized within-participant contrast across the four sensory conditions; the OA sway index was designated as the primary outcome because it jointly reflects the anteroposterior (AP) and mediolateral (ML) sway components. Accordingly, the “Means: Difference from constant (one sample case)” option under the *t*-test family was selected. Because no prior data were available for Al Balance, a medium standardized effect size (Cohen’s *d* = 0.50) was assumed according to Cohen’s conventions [15]; with a two-sided alpha level of 0.05 and 80% power, 34 participants were required. Allowing for an anticipated attrition rate of approximately 15% over the 7-day follow-up interval, the recruitment target was increased to 40 participants. This calculation addressed the sensory-condition hypotheses; no separate a priori calculation was performed for the reliability objective. For two sessions, a sample of 40 participants yields an expected 95% confidence interval (CI) width of approximately 0.23 for an intraclass correlation coefficient (ICC) of 0.80 [16]; because the precision of reliability estimates depends on sample size [17], all ICCs are reported with their 95% confidence intervals. The study was approved by the Non-Interventional Clinical Research Ethics Committee of Istanbul University-Cerrahpaşa (Approval No: 2024/71) and registered at ClinicalTrials.gov (NCT06224374). All participants provided written informed consent in accordance with the Declaration of Helsinki.

### 2.2. Protocol

**Figure 1:**
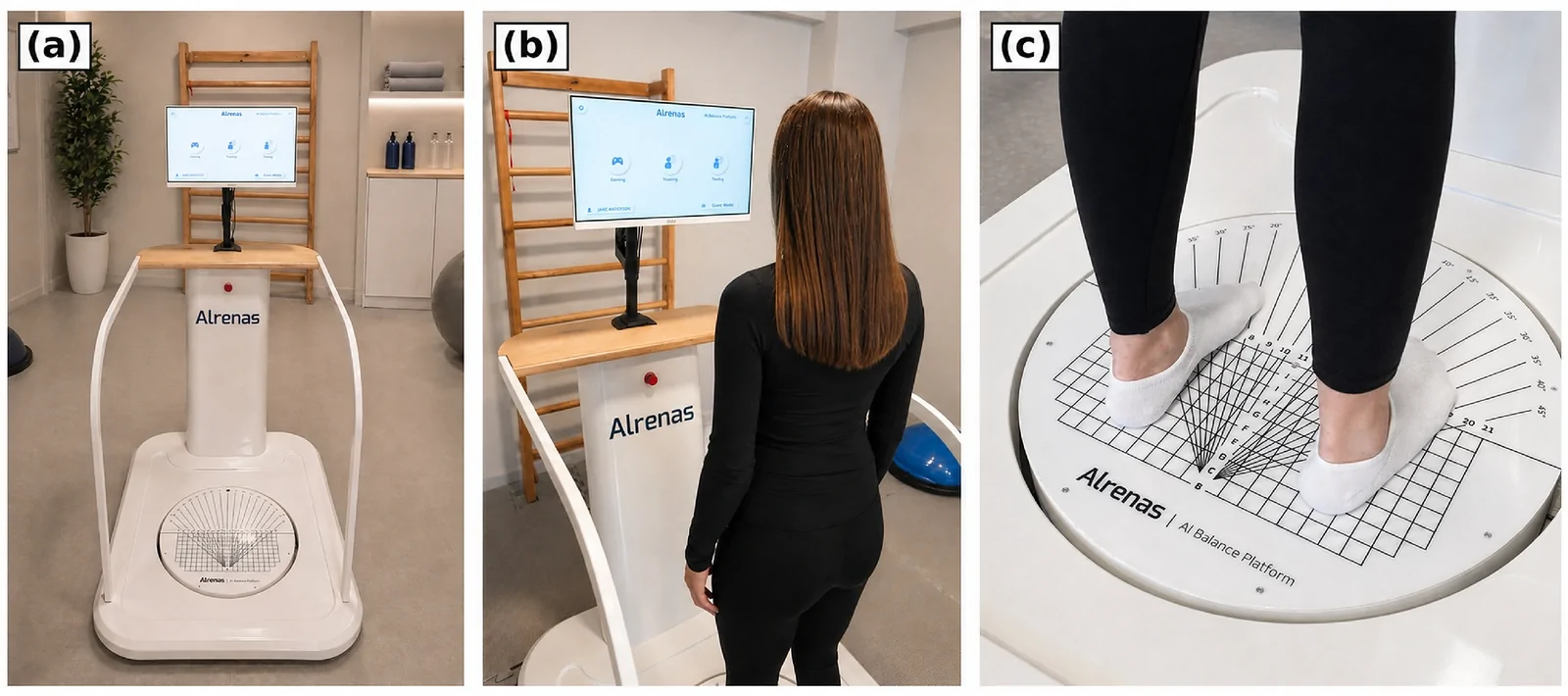
Overview of the Al Balance Stabilometric System and measurement configuration. (**a**) Integrated system comprising the static balance platform and touchscreen display. (**b**) Standardized participant positioning during postural control assessment. (**c**) Platform surface with foot-placement and directional reference markings used during testing.

The Al Balance Stabilometric System (Alrenas Technology Inc., Istanbul, Türkiye) is a medical device designed for the quantitative assessment and rehabilitation of postural control (Figure 1). The system features a static force platform equipped with four load cells and is integrated with a 24-inch all-in-one touchscreen computer, enabling real-time data acquisition, visualization, and analysis within a single unit. The platform is mechanically designed to ensure that only vertical forces are transmitted to the four load cells. Linear guiding elements are incorporated to resist lateral forces and prevent their transmission to the load cells. The load-bearing upper plate transfers forces to the load cells through unilateral point-contact interfaces, without rigid mechanical connections between the plate and the sensors. This configuration minimizes lateral loading on the sensors. No inertial sensors or optical cameras are used in the system. The system software derives AP and ML sway, OA stability scores, and directional control parameters from the load-sensor signals, with a sampling rate of at least 400 Hz according to the manufacturer [13]. Assessments were performed using Al Balance software version 2.0.5. In static-platform mode, the software estimates ML and AP displacement from differences between opposing load-sensor readings. These differences are normalized to total measured load and adjusted for platform geometry and participant-specific reference offsets. The resulting quantities represent platform-derived postural displacement estimates rather than directly measured whole-body center-of-mass coordinates. Sway indices quantify displacement variability around the mean position during a trial. Their calculation involves arithmetic averaging, subtraction of the trial mean, squaring of the resulting deviations, averaging of the squared deviations, and square-root extraction. Accordingly, the AP and ML sway indices represent root-mean-square deviations about the corresponding trial means. The OA sway index combines the squared deviations from both axes before averaging and square-root extraction. In the Postural Stability module, stability indices quantify displacement from the reference origin. The AP and ML indices are calculated by averaging the absolute displacement along each axis. The OA stability index is obtained by calculating the Euclidean magnitude of the two-dimensional displacement at each observation and then averaging these magnitudes over the trial. Thus, sway indices describe variability around the trial mean, whereas stability indices describe displacement from the reference origin. The outputs are expressed as device-specific, software-scaled displacement indices. Standardized assessment protocols include the Modified Clinical Test of Sensory Interaction on Balance (mCTSIB), Limits of Stability (LoS), Bilateral Comparison Test (BCT), and Postural Stability Test (PST), which are widely used protocols in postural control research [1,9,10,11]. The system also offers artificial intelligence-supported, game-based training modules, which are separate from the assessment modules and were not used in this study [13].

The Biodex Balance System SD (Model 950-302, Biodex Medical Systems, Shirley, NY, USA) was used as an established comparison instrument because it is widely used in clinical and research settings and offers protocols that are conceptually matched to the Al Balance sensory-condition and unilateral stance modules. It consists of a dynamic, multi-axial tilting platform with triaxial displacement sensors and an external monitor with a software interface. The device operates at a sampling rate of 40 Hz and includes standardized balance-assessment protocols including mCTSIB, LoS, and bilateral/unilateral stability assessments. The reliability of these protocols depends on the task, outcome, and population: in healthy adults, Dawson et al. reported ICCs of 0.83 for the LoS composite score and 0.75 for the overall mCTSIB score [9]; Parraca et al. reported ICCs of 0.69–0.80 for stability and fall-risk indices in physically active older adults [10]; Cachupe et al. reported that the reliability of BBS measures varied across test conditions [11]; and Peller et al. used the BBS as the reference instrument when validating the NIH Toolbox Standing Balance Test in community-dwelling older adults [12]. Like Al Balance, the Biodex System uses instrument-specific scoring algorithms and dedicated hardware components for data processing and storage.

Demographic characteristics of the participants, including age, sex, height, weight, and BMI, were recorded. Following a 5 min seated rest, all participants completed the mCTSIB, LoS, BCT, and PST protocols using the Al Balance System. Biodex assessment comprised the corresponding four sensory conditions implemented using the static Postural Stability mode, separate unilateral stance assessments of each limb, and the LoS test. The four sensory conditions and unilateral stance assessments were considered conceptually matched across systems at the task level, although the proprietary test modules and scoring algorithms were not identical. No additional Biodex assessment was treated as a standalone comparator for the Al Balance PST. The order of device administration was determined by simple randomization to minimize potential order and learning effects. Before data collection, a random sequence generated in Microsoft Excel (Microsoft Corp., Redmond, WA, USA) assigned each participant to one of two device orders (Al Balance first or Biodex first), without blocking or stratification, and the assigned order was applied at the participant’s first session. This resulted in 21 participants being tested on Al Balance first and 19 on the Biodex System first.

To evaluate test–retest reliability, participants completed a second session using only the Al Balance Stabilometric System after a 7-day interval. All assessments were performed by the same trained assessor to ensure procedural consistency. In each testing session, participants were given standardized verbal instructions and, before data collection, performed one unrecorded familiarization trial of each test (approximately 2.5 min per test and 10 min in total). Within each device, the tests were administered in the following order: mCTSIB, LoS, BCT, and PST (PST on Al Balance only); the four mCTSIB conditions were performed in the order listed in Section 2.3.1, and in the unilateral stance test the right leg was tested first. Each test consisted of three trials with a one-minute rest in between, and participants rested for 2 min between tests. Participants who completed the evaluations with the first device rested for approximately 5 min and performed the tests in the same order on the other device.

During all standing tests, participants stood in socks with their feet placed on the foot-placement grid of the platform (Figure 1c), so that foot position was standardized using the platform coordinates. In bipedal tests, the arms hung relaxed at the sides and the gaze was directed at the screen in front of the participant (Figure 1b). The assessor checked the position before each trial, and a trial was repeated if the participant moved the feet, touched the bars, or lost balance. The platform is equipped with protective bars described by the manufacturer [13]; throughout testing, the supervising physiotherapist stood next to the participant to ensure safety, and the testing environment was arranged to allow safe mounting and dismounting.

### 2.3. Balance Assessments

#### 2.3.1. Four-Condition Sensory Assessment (mCTSIB)

The mCTSIB is a widely used protocol designed to evaluate postural stability under different sensory conditions by selectively altering visual and somatosensory input. The test includes four conditions: (1) eyes open on a firm surface, (2) eyes closed on a firm surface, (3) eyes open on a foam surface, and (4) eyes closed on a foam surface. Eye closure removes visual input, and the foam surface reduces the reliability of somatosensory information from the feet and ankles; when both are combined, postural control relies more heavily on the remaining unaltered input, particularly vestibular information [1,4,5,9]. The test therefore quantifies how sway changes when visual and/or support-surface information is challenged, but it does not isolate or directly quantify visual, somatosensory, or vestibular contributions. A standardized foam cushion manufactured by Alrenas Technology Inc., measuring 55 cm in diameter and 10 cm in thickness, with a nominal density designation of 55 DNS (approximately 55 kg/m^3^) and designated foot markings, was used in conditions 3 and 4 to ensure proper foot placement; because foam properties can influence sway outcomes [18], the same cushion was used for all participants, in both sessions, and on both systems. Each condition was tested three times, with 30 s trials separated by one-minute rest intervals. AP, ML, and OA sway indices were recorded in each of the four conditions [10]. In the present study, the four sensory conditions were administered using the mCTSIB module of the Al Balance System, whereas on the Biodex System the corresponding four conditions were implemented using its static Postural Stability mode.

#### 2.3.2. Limits of Stability (LoS) Assessment

The LoS test assesses an individual’s ability to intentionally shift their center of gravity in multiple directions while maintaining postural control. Participants stood on a stable platform and were prompted via screen cues to shift their weight toward eight targets: front, front-right, right, back-right, back, back-left, left, and front-left. They were instructed to move their body weight toward each direction without lifting their feet or heels and then return to a central neutral position. Directional control for each movement was recorded, reflecting voluntary control of postural adjustments [9,11]. The test was target-directed: participants shifted their weight toward targets displayed at predefined positions by each system’s software rather than to their individual maximum voluntary excursion, so the outcome reflects directional control toward these targets and not the anatomical limits of stability. Al Balance assessments used the standard LoS configuration, without difficulty-based adjustment. The software provides predefined, direction-specific angular reference values that remain fixed across participants. These values define the displayed targets but do not cap the recorded excursion. The software records the maximum angular excursion achieved in each direction during the task, including movement beyond the predefined targets. Participant height is incorporated into the conversion of platform-derived displacement to angular excursion through an estimated center-of-gravity height. This conversion uses height normalization and an inverse trigonometric operation, with the resulting angles expressed in degrees. Diagonal angular excursions combine the AP and ML angular components using a Euclidean magnitude calculation. Consequently, the angular reference values are fixed, whereas the displacement corresponding to a given angular value varies with participant height. The two systems quantify LoS performance differently: the Biodex Balance System expresses directional control as a percentage-based score, whereas the Al Balance System scores each direction separately in degrees relative to predefined angular displacement limits. Because these outputs are based on different algorithms, scales, and computational definitions, LoS was not included in cross-device construct-validity analyses. LoS outcomes were interpreted within each device, and test–retest reliability was evaluated for Al Balance.

#### 2.3.3. Unilateral Stance Assessment (Bilateral Comparison Test, BCT)

The BCT evaluates static balance on one leg and is commonly used to compare postural stability between limbs. During this test, participants stood on one leg for 30 s with hands crossed over their opposite shoulders, performing three trials on each leg with eyes open. The task quantifies unilateral stability and potential asymmetries between the right and left limbs [10,11]; results are reported by side rather than by limb dominance. Compared with a conventional timed single-leg stance on the floor, which records only how long the position is maintained, the instrumented test quantifies the magnitude and direction (AP and ML) of sway while stance is maintained for a fixed duration, which can reveal differences between limbs in individuals who would reach the ceiling of a timed test. Unilateral stance was assessed using the BCT module of the Al Balance System, whereas on the Biodex System each limb was evaluated with separate unilateral stance assessments.

#### 2.3.4. Postural Stability Assessment (PST)

The PST is designed to quantify spontaneous sway during quiet standing. Participants stood on a stable platform, focusing on a screen in front of them while maintaining an upright posture for 30 s. The test was repeated three times, each separated by a one-minute rest period. Al Balance recorded AP, ML, and OA sway indices and the corresponding AP, ML, and OA stability indices, all derived by the system software from the load-sensor signals of the force platform. On the Biodex System, the static Postural Stability mode was used to implement the four sensory conditions rather than as a standalone PST; therefore, PST outcomes were retained for Al Balance test–retest reliability and descriptive within-device interpretation only and were not used to establish cross-device construct validity.

### 2.4. Statistical Analysis

The design, conduct, and reporting of the reliability and measurement-error analyses were guided by the COSMIN Risk of Bias tool for studies on reliability or measurement error, which explicitly covers performance-based measurement instruments [19]. Descriptive statistics, including means and standard deviations, were calculated to summarize the demographic characteristics of participants and the results obtained from each balance test protocol.

Because the BBS was regarded as an established comparator rather than a gold standard, validity was examined as hypothesis-testing construct validity [14]. Construct validity was evaluated by testing a priori hypotheses: that eye closure and a compliant surface would each increase sway scores on both systems, that the combination of eye closure and a compliant surface would produce the greatest deterioration, that the eyes-closed/foam condition would therefore be identified as the most challenging condition by both systems, and that within-participant change across the sensory conditions would progress in parallel between systems. The analyses were not designed to establish numerical equivalence or interchangeability between the two systems, and no such claim is made.

Cross-device validity analyses were restricted to assessments with conceptually matched task demands and clinically interpretable condition- or side-related response patterns. Accordingly, the four sensory conditions of the mCTSIB and the unilateral stance assessments of the BCT were included in the cross-device construct-validity analyses. PST and LoS outcomes were not included because the two systems generate these scores using different algorithms, scales, and computational definitions, precluding direct interpretation of the scores as measurements of the same underlying quantity. These modules were therefore evaluated only as device-specific outcomes and for Al Balance test–retest reliability. No Bland–Altman analysis, between-device intraclass correlation, concordance correlation coefficient, Deming regression, or other numerical interchangeability analysis was performed.

Because the Biodex static Postural Stability mode was used to implement the four sensory conditions rather than as a standalone PST, no cross-device PST analysis was performed. The Biodex eyes-open/firm mCTSIB condition was not treated as a numerical substitute for the Al Balance PST in the construct-validity analysis.

Within-device responsiveness to sensory manipulation was examined for the mCTSIB using 2 × 2 (vision × surface) repeated-measures analyses of variance (ANOVAs), conducted separately for each device and each axis, with partial eta squared (η_p_^2^) reported as the effect size. Because both within-subject factors had two levels, sphericity was not applicable to these models. Because Shapiro–Wilk tests indicated departures from normality in 16 of the 24 device × axis × condition cells, the a priori sensory hypotheses were additionally confirmed using Wilcoxon signed-rank tests on participant-level vision, surface, and vision × surface contrasts. Device-specific direction effects for LoS were examined with repeated-measures ANOVA and Greenhouse–Geisser correction where appropriate.

Cross-device convergence for the mCTSIB was assessed using repeated-measures correlations (rrm) with 95% confidence intervals, within-participant Spearman rank correlations between the two systems’ four-condition profiles summarized as the median (interquartile range) and percentage of positive profiles, agreement on the most challenging sensory condition, and standardized device × condition interactions. Repeated-measures correlations were derived from analysis of covariance with participant entered as a fixed factor and a common slope, following Bakdash and Marusich [20]. For unilateral stance, standardized device × side interactions were examined and participant-level normalized right–left asymmetry scores were compared using Spearman rank correlations. Because the systems produce scores on different scales, device × condition and device × side models were fitted to scores standardized within device; only the interaction terms were interpreted. Between-device Spearman correlations of participant-level sensory response magnitudes were adjusted for multiple testing using the Holm procedure. The test family was defined before the correlation analyses were performed as all nine correlations, i.e., three contrasts (vision, surface, and vision × surface) for each of three axes (AP, ML, and OA).

For the test–retest reliability analyses presented in Section 3.3, the session-level score for each mCTSIB, BCT, LoS, and PST outcome was calculated as the mean of three trials performed during the corresponding session. Relative reliability between the Day 1 and Day 7 session-level scores was evaluated using an intraclass correlation coefficient based on a two-way mixed-effects model, an absolute-agreement definition, and a single-measure form [ICC(A,1)]. The 95% CI for the ICC estimates were calculated using the ANOVA-based *F* method with the Satterthwaite approximation. ICC values were interpreted as poor (<0.50), moderate (0.50–0.75), good (>0.75–0.90), or excellent (>0.90) [21]. These categories describe the relative reliability of the measurements, not the participants’ balance performance, and were interpreted together with the 95% confidence intervals.

Potential systematic change between sessions was evaluated using the mean difference (Day 7 minus Day 1) and its 95% confidence interval. Absolute reliability was quantified using the standard error of measurement (SEM = SD_pooled_ × √(1 − ICC), where SD_pooled_ = √[(SD_Day1_^2^ + SD_Day7_^2^)/2]) and the minimal detectable change (MDC_95_ = SEM × 1.96 × √2) [22], expressed in the same units as the corresponding outcomes. MDC_95_ indicates the smallest change in a session score that exceeds measurement error with 95% confidence under this protocol; it does not by itself indicate a clinically important change. All reliability and measurement-error analyses were based on 40 paired observations, i.e., the Day 1 and Day 7 Al Balance session scores of the same 40 participants; no between-device reliability coefficient was calculated. All statistical analyses were performed using IBM SPSS Statistics version 28.0 (IBM Corp., Armonk, NY, USA). The Holm adjustment of the response-magnitude correlations and the median and interquartile range of the participant-level profile correlations were computed from the SPSS output in a spreadsheet. A *p*-value of less than 0.05 was considered statistically significant in all analyses.

## 3. Results

### 3.1. Participant Characteristics

A total of 40 healthy participants (23 women and 17 men) completed the study protocol. All participants attended both test sessions, and there were no dropouts or missing data. The majority of participants were right-side dominant (*n* = 36). The participants had a mean age of 22.62 ± 2.32 years, a mean height of 1.70 ± 0.09 m, a mean body weight of 64.30 ± 9.48 kg, and a mean BMI of 22.07 ± 1.91 kg/m^2^.

### 3.2. Construct Validity of the Al Balance System

Construct validity was evaluated in three steps. First, within-device responsiveness to sensory manipulation was examined for the four-condition sensory assessment (mCTSIB) using 2 × 2 (vision × surface) repeated-measures ANOVAs separately for each device. Second, device-specific results for unilateral stance, PST, and LoS were summarized without assuming numerical equivalence between systems. Third, cross-device construct validity was evaluated only for the conceptually matched four-condition sensory assessment and unilateral stance tasks using repeated-measures correlations, within-participant profiles, and standardized device × condition or device × side interactions. Detailed results are presented in Table 1, Table 2 and Table 3.

#### 3.2.1. Within-Device Sensory Responsiveness (Four-Condition Sensory Assessment, mCTSIB)

Both systems detected the expected effects of sensory manipulation (Table 1). For the Al Balance System, significant main effects of vision (OA sway: *F* = 71.60, *p* < 0.001, η_p_^2^ = 0.647) and surface (*F* = 130.80, *p* < 0.001, η_p_^2^ = 0.770), as well as a significant vision × surface interaction (*F* = 30.11, *p* < 0.001, η_p_^2^ = 0.436), were observed; the same pattern held for the AP and ML indices. The Biodex System likewise showed significant vision (*F* = 201.81, *p* < 0.001, η_p_^2^ = 0.838), surface (*F* = 36.58, *p* < 0.001, η_p_^2^ = 0.484), and interaction effects (*F* = 18.63, *p* < 0.001, η_p_^2^ = 0.323) for OA sway; the AP vision × surface interaction was also significant (*F* = 10.97, *p* = 0.002, η_p_^2^ = 0.220). In both systems, the eyes-open/firm condition produced the lowest and the eyes-closed/foam condition the highest mean sway scores. For OA sway, the Biodex mean increased from 0.41 ± 0.19 to 1.82 ± 0.65 across these conditions, while Al Balance increased from 0.034 ± 0.010 to 0.069 ± 0.018. These absolute values are device-specific and were not compared numerically.

Because Shapiro–Wilk tests indicated departures from normality in 16 of the 24 device × axis × condition cells, the three a priori sensory contrasts were re-tested non-parametrically. All 18 Wilcoxon signed-rank tests remained significant in the hypothesized direction; 17 reached *p* < 0.001 and the smallest effect, the Biodex vision × surface contrast in the AP axis, reached *p* = 0.001 (Table S2). The proportion of participants whose contrast moved in the predicted direction ranged from 72.5% to 100%, with the vision effect the most consistent on the Biodex System, where 100% of participants deteriorated with eye closure in both the OA and AP axes. The non-parametric results therefore agreed with the parametric analyses.

#### 3.2.2. Within-Device Effects: Unilateral Stance, Postural Stability, and Limits of Stability

For the unilateral stance assessment, the device-specific left and right scores are presented in Table 2; no within-device left–right difference reached statistical significance on either system (all *p* ≥ 0.144). PST outcomes are reported descriptively for Al Balance because the Biodex static Postural Stability mode was used to implement the four sensory conditions rather than as a standalone PST; the Biodex values in the PST rows of Table 2 therefore represent the same single eyes-open/firm quiet-stance measurement reported in Table 1, presented again for comparison rather than obtained from a repeated assessment. In the LoS test, directional performance differed significantly within each system. The direction effect was pronounced for Al Balance (Greenhouse–Geisser corrected F(2.51, 97.88) = 76.02, *p* < 0.001, η_p_^2^ = 0.661) and smaller for Biodex (F(5.45, 212.45) = 3.92, *p* = 0.001, η_p_^2^ = 0.091). Because PST and LoS use instrument-specific scoring definitions, these modules were not used to establish cross-device construct validity.

#### 3.2.3. Cross-Device Construct Validity

For the mCTSIB, cross-device convergence was supported at multiple levels (Table 3). Repeated-measures correlations between devices were moderate and positive (OA: r_rm_ = 0.598, 95% CI 0.470–0.700; AP: r_rm_ = 0.586, 95% CI 0.450–0.690; ML: r_rm_ = 0.513, 95% CI 0.370–0.630; all *p* < 0.001), and within-participant profiles were predominantly positive (median ρ = 0.716, interquartile range [IQR] 0.379–0.862, with 90.0% positive profiles for OA sway; 87.5% and 85.0% for AP and ML, respectively). The two systems identified the same most challenging sensory condition in 80.0% (OA), 60.0% (AP), and 70.0% (ML) of participants. Significant standardized device × condition interactions (OA: F(3, 117) = 20.85, *p* < 0.001, η_p_^2^ = 0.348; AP: F(3, 117) = 20.80, *p* < 0.001, η_p_^2^ = 0.348; ML: F(3, 117) = 10.47, *p* < 0.001, η_p_^2^ = 0.212) indicated that the relative magnitude of sensory-condition effects differed between systems. For the BCT, device × side interactions were non-significant (OA: F(1, 39) = 0.35, *p* = 0.559, η_p_^2^ = 0.009; AP: F(1, 39) = 0.00, *p* = 0.983, η_p_^2^ < 0.001; ML: F(1, 39) = 0.03, *p* = 0.868, η_p_^2^ = 0.001). These non-significant interactions indicate only that the group-level right–left pattern did not differ between devices; they do not establish measurement equivalence or interchangeability of individual asymmetry values. Participant-level asymmetry correlations were weak and non-significant (OA ρ = −0.091, *p* = 0.575; AP ρ = −0.093, *p* = 0.568; ML ρ = −0.275, *p* = 0.086), and the two devices assigned the same direction of asymmetry in only 27.5–45.0% of participants (OA 45.0%, AP 42.5%, ML 27.5%), i.e., in fewer than half of the participants on every axis.

Greenhouse–Geisser correction for violations of sphericity did not alter the significance of any device × condition interaction (Table S1).

Participant-level response magnitudes were positive for every contrast on both systems. Between-device Spearman correlations of these magnitudes were weak (Table S3); the strongest association, for the vision effect in the OA axis (ρ = 0.411, uncorrected *p* = 0.008), did not remain significant after Holm adjustment across nine tests (adjusted *p* = 0.076), and three of the nine point estimates were negative. These two analyses address different questions: the repeated-measures correlation evaluates whether within-participant changes across all four mCTSIB conditions are consistent between devices, whereas the Spearman correlations in Table S3 quantify between-device agreement on the magnitude of individual sensory effects. Because response magnitudes are difference scores, they accumulate the measurement error of both conditions from which they are derived; the weak magnitude correlations therefore do not contradict the moderate repeated-measures correlations.

### 3.3. Test–Retest Reliability of the Al Balance Stabilometric System

#### 3.3.1. Four-Condition Sensory Assessment (mCTSIB)

Across the four sensory conditions of the mCTSIB, the Al Balance Stabilometric System demonstrated moderate to excellent test–retest reliability, with predominantly good values (ICC(A,1) = 0.678–0.909; Table 4). Reliability was good for eight of the twelve outcomes (ICC(A,1) = 0.764–0.872), excellent for the eyes-closed/foam AP index (ICC(A,1) = 0.909), and moderate for three outcomes: the eyes-open/firm ML (0.734), eyes-open/foam AP (0.678), and eyes-closed/foam ML (0.703) indices. SEM values ranged from 0.003 to 0.007, and MDC_95_ values ranged from 0.009 to 0.020. Small between-session increases were observed for the eyes-closed/foam AP and OA indices; these differences were below the corresponding MDC_95_ values. These coefficients describe the repeatability of Al Balance scores within the device; they do not indicate individual-level agreement with the Biodex System (Section 3.2.3).

#### 3.3.2. Unilateral Stance Assessment (BCT)

The Al Balance Stabilometric System demonstrated good to excellent test–retest reliability for the BCT (ICC(A,1) = 0.759–0.929; Table 4). The ML sway indices showed excellent reliability on both sides (ICC(A,1) = 0.924 for the left and 0.929 for the right leg), whereas the AP and OA sway indices showed good reliability (ICC(A,1) = 0.759–0.888). SEM values ranged from 0.003 to 0.010, and MDC_95_ values ranged from 0.008 to 0.027. A small between-session increase was observed for the right-leg OA sway index; this difference was below the corresponding MDC_95_. Although the right- and left-limb BCT scores were repeatable within Al Balance, the individual asymmetry scores derived from them converged weakly with those of the Biodex System (Section 3.2.3).

#### 3.3.3. Limits of Stability Assessment

The LoS outcomes demonstrated good to excellent test–retest reliability (ICC(A,1) = 0.873–0.936; Table 5). The composite score and six of the eight directional scores showed excellent reliability, with ICC(A,1) values ranging from 0.902 to 0.936, while the right and left directional scores demonstrated good reliability (ICC(A,1) = 0.888, 95% CI: 0.803–0.939, and 0.873, 95% CI: 0.777–0.930, respectively). SEM values ranged from 0.182 to 0.400, and MDC_95_ values ranged from 0.504 to 1.109. The 95% confidence intervals for the mean differences included zero for seven of the nine LoS outcomes; a small increase was observed for the front-right direction (mean difference: 0.171; 95% CI: 0.046–0.295), and a small decrease for the back direction (mean difference: −0.131; 95% CI: −0.234 to −0.029).

#### 3.3.4. Postural Stability Assessment

The PST outcomes demonstrated good to excellent test–retest reliability (ICC(A,1) = 0.866–0.946; Table 5). Among the sway indices, AP and OA sway showed excellent reliability (ICC(A,1) = 0.915 and 0.903, respectively), whereas ML sway showed good reliability (ICC(A,1) = 0.866). All three stability indices demonstrated excellent reliability (ICC(A,1) = 0.910–0.946). SEM values ranged from 0.003 to 0.004 for the sway indices and from 0.024 to 0.038 for the stability indices; the corresponding MDC_95_ values ranged from 0.008 to 0.012 and from 0.067 to 0.104, respectively. The 95% confidence intervals for the mean differences included zero for all PST outcomes except the OA stability index, which showed a small increase between sessions (mean difference: 0.026; 95% CI: 0.011–0.041).

## 4. Discussion

This study evaluated the hypothesis-based construct validity and test–retest reliability of the Al Balance Stabilometric System in healthy young adults, using the Biodex Balance System SD as an established comparison instrument. Three main findings emerged. First, both systems captured the expected effects of visual occlusion and surface compliance in the mCTSIB, and cross-device convergence was supported by moderate repeated-measures correlations, predominantly positive within-participant profiles, and agreement on the most challenging sensory condition. Second, significant device × condition interactions and weak between-device correlations of individual limb asymmetry indicated that the two systems should not be considered numerically interchangeable; for unilateral stance, convergence evidence was limited. Third, Al Balance demonstrated outcome-dependent test–retest reliability, ranging from moderate to excellent and predominantly good to excellent (ICC(A,1) = 0.678–0.946). Together, the results support construct validity most strongly for sensory-condition responsiveness, with limited evidence for unilateral stance, while PST and LoS should be interpreted as device-specific modules.

### 4.1. Validity: Convergence Between the Al Balance and Biodex Systems

Both devices reproduced the expected sensory-interaction pattern of the mCTSIB, with greater sway during visual occlusion, surface compliance, and their combination. This indicates that Al Balance scores respond in the expected direction to the sensory manipulations, which supports construct validity but does not demonstrate numerical agreement with the BBS. The pattern is consistent with sensory reweighting: when vision is removed or support-surface information becomes less reliable, postural control relies more on the remaining inputs, and center-of-pressure sway and postural strategies change accordingly [4,5]. Recent studies have examined postural balance in young people with intellectual disabilities [23,24]; in one of these, eye closure increased center-of-pressure velocity [23], consistent with the visual effect observed here. Because their populations and tasks differ from those of the present study, these studies provide physiological context rather than direct evidence on Al Balance. Cross-device convergence was further supported by moderate repeated-measures correlations and predominantly positive within-participant condition profiles, and both systems frequently identified the same most challenging condition. In unilateral stance, the non-significant device × side interactions should not be interpreted as evidence of equivalence, because they do not test whether the two devices measure the same individual asymmetry. At the participant level, asymmetry correlations were weak, and the two devices assigned the same direction of asymmetry in only 27.5–45.0% of participants; individual asymmetry scores should therefore not be transferred between systems. Similarly, the weak between-device correlations of participant-level response magnitudes (Table S3) are not unexpected, because difference scores compound the measurement error of the two conditions from which they are formed; they do not contradict the repeated-measures correlations, which are estimated across all four sensory conditions simultaneously. PST and LoS were not used for cross-device construct-validity claims because their numerical outputs are based on non-equivalent algorithms and scales.

In contrast, the significant device × condition interactions and the markedly larger vision effect observed for the Biodex System (η_p_^2^ = 0.838 vs. 0.647 for OA sway) indicate that the magnitude of condition effects, particularly when visual input is removed, differs substantially between devices. Several factors could contribute to this pattern; because none of them was tested directly in the present study, they should be regarded as possible explanations rather than established causes. Occluding vision shifts sensory weighting toward proprioceptive and vestibular inputs and increases both the amplitude and the frequency content of postural sway [4], so differences in how devices capture sway may become larger as sway increases. The two systems also differ in hardware: the Al Balance System uses a rigid static plate with four load sensors, whereas the Biodex platform, even in its static configuration, is a multi-axial tilting surface [11], and each system computes its scores with its own proprietary algorithms. Their nominal sampling rates also differ (400 Hz vs. 40 Hz); current standardization guidelines for clinical stabilometry recommend a minimum sampling frequency of 50 Hz [25], and the 40 Hz rate of the Biodex System is slightly below this recommendation. However, a higher nominal sampling rate does not by itself indicate greater measurement accuracy, because the information retained also depends on filtering and signal processing, which are not fully documented for either system. Because hardware design, sampling frequency, filtering, and proprietary scoring algorithms cannot be fully matched between the two platforms, these factors are likely to have contributed to the cross-device discrepancies observed here, including the larger vision effect on the Biodex System and the weak agreement of individual asymmetry scores; their separate contributions could not be isolated in the present design. In practice, scores obtained from the two systems should not be compared directly or used interchangeably, particularly under eyes-closed conditions and for individual limb-asymmetry profiles; repeated measurements should be made with the same device and interpreted against its outcome-specific measurement error.

### 4.2. Reliability of the Al Balance System

The Al Balance System demonstrated outcome-dependent test–retest reliability, ranging from moderate to excellent: predominantly good for the mCTSIB and BCT outcomes, and predominantly excellent for the LoS and PST outcomes. This is a within-device property: it shows that Al Balance scores are reproducible across sessions, but it does not guarantee individual-level agreement with the Biodex System, with which individual sensory-response magnitudes and asymmetry scores converged weakly (Section 4.1). Not all sensory-challenged conditions were equally reliable: three mCTSIB outcomes showed moderate reliability, and some ICC confidence intervals were wide (for the eyes-open/foam AP index, the lower bound was 0.475), so point estimates should not be over-interpreted. In contrast, the ML sway indices of the BCT were among the strongest outcomes (ICC(A,1) up to 0.929 with an MDC_95_ as low as 0.008). The use of three trials per protocol may have contributed to this consistency, in line with evidence that averaging repeated measurements improves the reliability of center-of-pressure-based parameters [26]. For the LoS test, the composite score and most directional scores showed excellent reliability, whereas the right and left directions showed good reliability, a direction-dependent variation suggesting that the repeatability of intentional weight-shifting may not be uniform across movement directions. The PST demonstrated excellent reliability for AP and OA sway and for all stability indices, while ML sway showed good reliability; reliability should therefore be interpreted separately for each outcome rather than generalized uniformly across the entire protocol. These coefficients are within the range of values reported for other systems: for the Biodex SD, ICCs of 0.83 for the LoS composite and 0.75 for the OA mCTSIB score have been reported in healthy adults [9], and 0.69–0.80 for stability and fall-risk indices in physically active older adults [10], compared with 0.930 (LoS composite) and 0.834–0.846 (OA mCTSIB scores) for the Al Balance System in the present study. Similarly, the Satel 40 Hz force platform demonstrated good to excellent test–retest reliability with a maximum ICC of 0.874 [1], and comparable coefficients have been reported for the Biodex Balance System in active adults [11] and the Wii Balance Board [2,3], although such comparisons should be interpreted cautiously given differences in populations, protocols, and ICC models, and they do not indicate that one device is more reliable than another. A recent force-platform study in adolescents, using a 12-week interval, likewise found that test–retest reliability varied markedly across postural outcomes and was considerably lower in unipedal than in bipedal stance [27]; the higher unipedal reliability in the present study may partly reflect the shorter interval and the averaging of three trials, and underlines the need to interpret reliability separately for each outcome and protocol. The moderate coefficients observed for three directional indices (eyes-open/firm ML, eyes-open/foam AP, and eyes-closed/foam ML) may reflect the greater trial-to-trial variability of directional sway components, particularly on compliant support surfaces. Because ICC values depend on between-participant variability, the relatively homogeneous healthy young sample is not expected to have inflated the coefficients; however, reliability and measurement error may differ in older adults or patients with larger and more variable sway, so these estimates should not be generalized to such populations [21,22]. With 40 participants, several confidence intervals were wide, and their lower bounds should be considered when judging the minimum plausible reliability of an outcome [17]. The outcome-specific SEM and MDC_95_ values indicate how large a change in a three-trial session score must be to exceed measurement error under this protocol [6]; exceeding the MDC_95_ does not by itself establish that a change is clinically important or attributable to an intervention. Small systematic between-session changes were identified for a minority of outcomes: two eyes-closed/foam mCTSIB indices, the right-leg OA BCT sway index, the LoS front-right and back directions, and the PST OA stability index. These differences may reflect familiarization, adaptation to the testing procedure, or direction-specific measurement variability; however, because they were small, well below the corresponding MDC_95_ values, and evaluated without adjustment for multiple comparisons, they should be interpreted cautiously rather than as definitive evidence of a learning effect.

### 4.3. Clinical and Technological Implications

The Al Balance System combines balance assessment with single-unit touchscreen operation, integrated data visualization, and separate AI-supported training modules. However, the present study evaluated only selected measurement properties of the assessment modules in healthy young adults; usability, clinical utility, safety in patients, and the effectiveness of the training modules were not assessed. The reported SEM and MDC_95_ values may help users judge whether a change in an individual’s Al Balance score exceeds the measurement error observed under this protocol [6], but corresponding values need to be established in the populations of interest. Individual asymmetry metrics derived from the BCT require particular caution: although the right- and left-limb scores from which they are derived were reliable within Al Balance, the two devices assigned the same direction of asymmetry in only 27.5–45.0% of participants, so side-to-side differences should not be used to guide clinical decisions, such as identifying the weaker limb, until their validity has been established in the population of interest. If the system is translated into clinical practice, it may be relevant for individuals with balance impairments of different origins, for example, age-related, neurological, vestibular [7], or musculoskeletal. In any such population, validity and reliability would need to be re-examined, together with practical safety aspects such as mounting and dismounting the platform, the use of the protective bars, and supervision during the most challenging sensory conditions, such as standing on foam with the eyes closed.

### 4.4. Limitations

This study has several limitations. First, all measurement-property findings, including construct validity, test–retest reliability, SEM, and MDC_95_, were obtained in healthy young adults aged 18–25 years and cannot be generalized to older adults or to patients with vestibular or neuromuscular disorders or an increased risk of falling, in whom sway is typically larger and more variable and measurement properties may differ. Second, because the two systems generate PST and LoS scores using different algorithms and scales, cross-device construct validity could be evaluated only for the conceptually matched four-condition sensory assessment (mCTSIB) and unilateral stance tasks. Third, although the mCTSIB demonstrated convergence in response patterns, significant device × condition interactions show that numerical scores remain device-specific. Fourth, individual asymmetry convergence was weak, so BCT-derived asymmetry metrics should be interpreted cautiously and should not be transferred across systems; the good within-device reliability of the right- and left-limb scores from which they are derived does not compensate for this limited individual-level agreement. Fifth, the sample size was calculated for the sensory-condition hypotheses rather than for the reliability objective, and some ICC estimates were imprecise (e.g., 95% CI 0.475–0.814 for the eyes-open/foam AP index). Sixth, although the measurement principles, mathematical operations, score interpretation, sampling rate, and software version are described, the exact proprietary equations, scaling coefficients, and implementation constants are not disclosed. This restricts independent reproduction of the software calculations. Detailed hardware-level filtering and load-sensor gain-calibration specifications were also unavailable for verification. These limitations should be considered when interpreting reproducibility and cross-device comparability. Finally, although device order was randomized, a familiarization trial was performed, and rest periods were provided, same-day testing on the two systems may have introduced minor fatigue or learning effects. In addition, adherence to the instruction to avoid additional task-specific practice between sessions was assessed through verbal self-report at the beginning of the Day 7 session and was not independently verified. Therefore, the possibility of unreported practice cannot be completely excluded.

### 4.5. Future Directions

Future studies should validate the system in older adults and in clinical populations with balance impairments, where measurement properties, feasibility, and safety may differ. Developing condition-specific calibration or conversion equations would improve cross-device comparability of scores, particularly under eyes-closed conditions, and responsiveness studies are needed to establish minimal clinically important differences. Randomized trials investigating the effectiveness of the system’s AI-based, game-supported training modules on rehabilitation outcomes are also warranted.

## 5. Conclusions

The Al Balance Stabilometric System demonstrated outcome-dependent test–retest reliability in healthy young adults, ranging from moderate to excellent and predominantly good to excellent. This within-device repeatability does not imply individual-level agreement with the Biodex System. Hypothesis-based construct validity was supported most strongly for sensory-condition responsiveness: both systems captured the expected hierarchy of mCTSIB challenges and showed parallel within-participant changes, whereas evidence for unilateral stance was limited. Significant device × condition interactions and weak convergence of individual asymmetry scores indicate that Al Balance and Biodex numerical outputs are device-specific and should not be used interchangeably. These findings support the use of Al Balance for describing sensory-condition-related changes in postural control in healthy young adults, with repeated measurements made on the same device; its measurement properties and safety in older adults and in patients with vestibular, neuromuscular, or other balance impairments, including those at increased risk of falling, remain to be established.

## Figures and Tables

**Table 1:** Modified Clinical Test of Sensory Interaction on Balance: condition scores and within-device sensory responsiveness (*n* = 40).

Device	Axis	EO/Firm	EC/Firm	EO/Foam	EC/Foam	Vision	Surface	Vision × Surface
Al Balance	Overall	0.034 ± 0.010	0.045 ± 0.014	0.048 ± 0.011	0.069 ± 0.018	71.60 (0.647)	130.80 (0.770)	30.11 (0.436)
	AP	0.028 ± 0.010	0.036 ± 0.012	0.036 ± 0.009	0.056 ± 0.017	99.33 (0.718)	79.77 (0.672)	28.05 (0.418)
	ML	0.020 ± 0.008	0.024 ± 0.009	0.028 ± 0.009	0.043 ± 0.013	37.50 (0.490)	96.74 (0.713)	24.86 (0.389)
Biodex	Overall	0.41 ± 0.19	1.26 ± 0.57	0.51 ± 0.16	1.82 ± 0.65	201.81 (0.838)	36.58 (0.484)	18.63 (0.323)
	AP	0.28 ± 0.14	0.98 ± 0.42	0.35 ± 0.11	1.28 ± 0.39	247.06 (0.864)	20.88 (0.349)	10.97 (0.220)
	ML	0.21 ± 0.13	0.66 ± 0.53	0.28 ± 0.11	1.14 ± 0.47	123.08 (0.759)	30.13 (0.436)	21.54 (0.356)

EO, eyes open; EC, eyes closed; AP, anteroposterior; ML, mediolateral; η_p_^2^, partial eta squared, SD, standard deviation; ANOVA, analysis of variance; df, degrees of freedom. The four sensory-condition columns present mean ± SD; the three right-hand columns present F (η_p_^2^) for the corresponding repeated-measures ANOVA effect. Higher scores indicate greater postural sway, i.e., poorer balance. Biodex scores are Stability Index values as displayed by the device; Al Balance scores are the raw output of that system. The two scales are not numerically comparable. All ANOVA effects have df = 1, 39, and all main effects and interactions were statistically significant (all *p* ≤ 0.002). Because both within-subject factors have two levels, sphericity is not applicable and no correction was required. Wilcoxon signed-rank tests on the participant-level vision, surface and interaction contrasts confirmed every effect non-parametrically for both devices in all three axes (all *p* < 0.002), with 72.5–100% of participants responding in the hypothesized direction. The eyes-closed/foam condition produced the highest and the eyes-open/firm condition the lowest mean score for both devices in all three axes.

**Table 2:** Unilateral stance, postural stability, and limits of stability: device-specific scores and within-device effects (*n* = 40).

Test	Level	Al Balance (Mean ± SD)	Biodex (Mean ± SD)	Device-Specific Effect/Note
Unilateral stance (BCT)	Overall, left	0.079 ± 0.023	1.02 ± 0.44	—
	Overall, right	0.081 ± 0.018	1.08 ± 0.56	—
	AP, left	0.064 ± 0.016	0.73 ± 0.30	—
	AP, right	0.065 ± 0.017	0.78 ± 0.42	—
	ML, left	0.049 ± 0.014	0.65 ± 0.31	—
	ML, right	0.049 ± 0.011	0.76 ± 0.42	—
Postural stability (PST)	AP	0.278 ± 0.119	0.28 ± 0.14	Descriptive only; cross-device validity not assessed
	ML	0.190 ± 0.076	0.21 ± 0.13	—
	Overall	0.373 ± 0.153	0.41 ± 0.19	—
Limits of stability (LoS)	Front	6.77 ± 1.05	64.65 ± 18.19	Al Balance: F(2.51, 97.88) = 76.02, *p* < 0.001, η_p_^2^ = 0.661
	Front-right	7.67 ± 1.03	53.98 ± 16.36	Biodex: F(5.45, 212.45) = 3.92, *p* = 0.001, η_p_^2^ = 0.091
	Right	7.15 ± 1.15	61.70 ± 16.86	—
	Back-right	5.73 ± 1.03	57.48 ± 17.24	—
	Back	4.18 ± 0.75	64.35 ± 17.85	—
	Back-left	5.64 ± 1.05	56.45 ± 16.76	—
	Left	6.90 ± 1.06	62.17 ± 14.57	—
	Front-left	7.34 ± 1.15	56.73 ± 14.18	—

BCT, Bilateral Comparison Test; PST, Postural Stability Test; LoS, Limits of Stability; AP, anteroposterior; ML, mediolateral, SD, standard deviation; η_p_^2^, partial eta squared; ANOVA, analysis of variance. Values are presented in each device’s own output scale and are not numerically comparable across systems. For LoS, higher scores denote better performance; for BCT and PST, higher scores denote poorer performance. No within-device left–right difference reached significance on either system in the BCT (all *p* ≥ 0.144). The LoS direction effects were examined separately within each device using repeated-measures ANOVA with Greenhouse–Geisser correction. PST values are presented descriptively. The Biodex PST entries are the eyes-open/firm quiet-stance scores reproduced from Table 1, obtained with the Biodex static Postural Stability mode, which was used to implement the four sensory conditions rather than as a standalone PST; PST was therefore not included in cross-device construct-validity analyses.

**Table 3:** Cross-device construct validity for mCTSIB and unilateral stance assessments (*n* = 40).

Test Block	Axis/Level	Within-Participant Convergence	Individual Profile Similarity	Device × Condition Interaction	Support
mCTSIB	Overall	r_rm_ = 0.598 [0.470, 0.700] ***	Mdn ρ = 0.716 [0.379, 0.862]	F(3, 117) = 20.85, η_p_^2^ = 0.348 ***	Yes
	AP	r_rm_ = 0.586 [0.450, 0.690] ***	Mdn ρ = 0.632 [0.400, 0.800]	F(3, 117) = 20.80, η_p_^2^ = 0.348 ***	Yes
	ML	r_rm_ = 0.513 [0.370, 0.630] ***	Mdn ρ = 0.632 [0.316, 0.804]	F(3, 117) = 10.47, η_p_^2^ = 0.212 ***	Yes
	Hierarchy	Same most challenging condition	80.0% (Overall), 60.0% (AP), 70.0% (ML)	—	Yes
Unilateral (BCT)	Overall	Asymmetry ρ = −0.091, *p* = 0.575	Sign agreement 45.0%	F(1, 39) = 0.35, *p* = 0.559, η_p_^2^ = 0.009	Limited
	AP	Asymmetry ρ = −0.093, *p* = 0.568	Sign agreement 42.5%	F(1, 39) = 0.00, *p* = 0.983, η_p_^2^ < 0.001	Limited
	ML	Asymmetry ρ = −0.275, *p* = 0.086	Sign agreement 27.5%	F(1, 39) = 0.03, *p* = 0.868, η_p_^2^ = 0.001	Limited

r_rm_, repeated-measures correlation [20], estimated from the four sensory conditions nested within 40 participants, with the 95% confidence interval in brackets; Mdn ρ, median of the participant-level Spearman correlations between the two devices’ four-condition profiles, with the interquartile range in brackets; sign agreement, percentage of participants for whom both devices returned a unilateral asymmetry of the same sign. Asymmetry was calculated as (right − left)/[(right + left)/2]. Device × condition and device × side interactions were tested on scores standardized within device. Uncorrected degrees of freedom are shown; Greenhouse–Geisser correction for the four-level mCTSIB condition factor did not alter significance (Table S1); BCT side has two levels and does not require a sphericity correction. In the hierarchy row, the percentages denote the proportion of participants for whom both devices identified the same most challenging sensory condition. Limited: group-level side patterns did not differ between devices, but individual asymmetry scores converged weakly; a non-significant device × side interaction is not evidence of equivalence. The table evaluates construct convergence rather than numerical equivalence or interchangeability, η_p_^2^, partial eta squared; ρ, Spearman’s rank correlation coefficient; AP, anteroposterior; ML, mediolateral; BCT, Bilateral Comparison Test; mCTSIB, Modified Clinical Test of Sensory Interaction on Balance. *** *p* < 0.001.

**Table 4:** Test–retest reliability and measurement error of mCTSIB and BCT outcomes measured with the Al Balance Stabilometric System (*n* = 40).

Outcome	Day 1 Mean ± SD (Min–Max)	Day 7 Mean ± SD (Min–Max)	Mean Difference (95% CI)	ICC(A,1) (95% CI)	SEM	MDC_95_
**mCTSIB**						
Firm, EO–AP	0.028 ± 0.010 (0.013–0.067)	0.028 ± 0.011 (0.010–0.067)	0.000 (−0.002, 0.002)	0.849 (0.736, 0.916)	0.004	0.012
Firm, EO–ML	0.020 ± 0.008 (0.010–0.043)	0.022 ± 0.011 (0.010–0.053)	0.001 (−0.001, 0.004)	0.734 (0.558, 0.848)	0.005	0.013
Firm, EO–OA	0.034 ± 0.010 (0.020–0.077)	0.036 ± 0.013 (0.020–0.080)	0.002 (−0.000, 0.004)	0.846 (0.734, 0.915)	0.005	0.013
Firm, EC–AP	0.036 ± 0.012 (0.020–0.073)	0.036 ± 0.013 (0.020–0.073)	0.000 (−0.002, 0.002)	0.872 (0.774, 0.929)	0.004	0.012
Firm, EC–ML	0.024 ± 0.009 (0.010–0.050)	0.025 ± 0.012 (0.010–0.063)	0.001 (−0.002, 0.003)	0.764 (0.603, 0.867)	0.005	0.014
Firm, EC–OA	0.045 ± 0.014 (0.020–0.080)	0.045 ± 0.015 (0.017–0.080)	0.000 (−0.002, 0.003)	0.840 (0.722, 0.911)	0.006	0.016
Foam, EO–AP	0.036 ± 0.009 (0.013–0.060)	0.037 ± 0.009 (0.020–0.063)	0.001 (−0.001, 0.004)	0.678 (0.475, 0.814)	0.005	0.015
Foam, EO–ML	0.028 ± 0.009 (0.017–0.053)	0.028 ± 0.008 (0.013–0.050)	0.001 (−0.001, 0.002)	0.858 (0.751, 0.921)	0.003	0.009
Foam, EO–OA	0.048 ± 0.011 (0.027–0.077)	0.049 ± 0.011 (0.033–0.080)	0.001 (−0.001, 0.003)	0.834 (0.713, 0.908)	0.005	0.013
Foam, EC–AP	0.056 ± 0.017 (0.033–0.103)	0.059 ± 0.017 (0.037–0.100)	0.002 (0.000, 0.005)	0.909 (0.838, 0.950)	0.005	0.014
Foam, EC–ML	0.043 ± 0.013 (0.023–0.087)	0.042 ± 0.012 (0.023–0.070)	−0.001 (−0.004, 0.002)	0.703 (0.510, 0.830)	0.007	0.019
Foam, EC–OA	0.069 ± 0.018 (0.033–0.117)	0.072 ± 0.018 (0.037–0.117)	0.003 (0.000, 0.007)	0.842 (0.727, 0.912)	0.007	0.020
**BCT**						
Left–AP	0.064 ± 0.016 (0.037–0.107)	0.066 ± 0.020 (0.033–0.113)	0.002 (−0.002, 0.006)	0.771 (0.614, 0.871)	0.009	0.024
Left–ML	0.049 ± 0.014 (0.033–0.097)	0.049 ± 0.014 (0.033–0.097)	0.000 (−0.001, 0.002)	0.924 (0.864, 0.959)	0.004	0.010
Left–OA	0.079 ± 0.023 (0.053–0.183)	0.081 ± 0.023 (0.043–0.160)	0.003 (−0.001, 0.007)	0.849 (0.738, 0.916)	0.009	0.025
Right–AP	0.065 ± 0.017 (0.037–0.103)	0.067 ± 0.020 (0.037–0.133)	0.003 (−0.000, 0.005)	0.888 (0.803, 0.939)	0.006	0.017
Right–ML	0.049 ± 0.011 (0.020–0.073)	0.049 ± 0.011 (0.020–0.070)	0.000 (−0.002, 0.001)	0.929 (0.872, 0.961)	0.003	0.008
Right–OA	0.081 ± 0.018 (0.047–0.117)	0.085 ± 0.022 (0.047–0.133)	0.004 (0.000, 0.009)	0.759 (0.597, 0.863)	0.010	0.027

Values are mean ± standard deviation (SD) with range (minimum–maximum), reported in the raw Al Balance output scale. Mean difference = Day 7 − Day 1; 95% confidence intervals (CIs) were calculated using the paired-samples *t* distribution. ICC(A,1) denotes a two-way mixed-effects, absolute-agreement, single-measure intraclass correlation coefficient based on session-level scores, each defined as the mean of three trials. SEM = SD_pooled_ × √[1 − ICC(A,1)], where SD_pooled_ = √[(SD_Day 1_^2^ + SD_Day 7_^2^)/2]; MDC_95_ = 1.96 × √2 × SEM. mCTSIB, Modified Clinical Test of Sensory Interaction on Balance; BCT, Bilateral Comparison Test; EO, eyes open; EC, eyes closed; AP, anteroposterior; ML, mediolateral; OA, overall; SEM, standard error of measurement; MDC_95_, minimal detectable change at the 95% confidence level. All estimates are based on 40 paired observations. Values are rounded to three decimals; all calculations used unrounded data.

**Table 5:** Test–retest reliability and measurement error of Limits of Stability and Postural Stability Test outcomes measured with the Al Balance Stabilometric System (*n* = 40).

Outcome	Day 1 Mean ± SD (Min–Max)	Day 7 Mean ± SD (Min–Max)	Mean Difference (95% CI)	ICC(A,1) (95% CI)	SEM	MDC_95_
**LoS**						
Composite	6.325 ± 0.682 (4.513–7.493)	6.400 ± 0.696 (4.613–7.533)	0.075 (−0.005, 0.155)	0.930 (0.875, 0.962)	0.182	0.504
Front	6.769 ± 1.049 (4.250–8.480)	6.872 ± 1.195 (4.120–8.477)	0.103 (−0.049, 0.255)	0.909 (0.837, 0.950)	0.340	0.943
Front-right	7.671 ± 1.025 (4.667–8.833)	7.841 ± 1.072 (4.767–9.267)	0.171 (0.046, 0.295)	0.920 (0.857, 0.956)	0.296	0.822
Right	7.148 ± 1.146 (4.267–9.457)	7.290 ± 1.012 (5.347–9.597)	0.142 (−0.018, 0.301)	0.888 (0.803, 0.939)	0.361	1.001
Back-right	5.731 ± 1.031 (3.000–7.567)	5.654 ± 0.987 (2.967–7.500)	−0.077 (−0.219, 0.066)	0.902 (0.826, 0.947)	0.316	0.875
Back	4.180 ± 0.748 (2.637–5.500)	4.049 ± 0.846 (2.107–5.500)	−0.131 (−0.234, −0.029)	0.909 (0.838, 0.950)	0.241	0.668
Back-left	5.635 ± 1.052 (3.300–7.533)	5.571 ± 1.165 (3.100–7.500)	−0.065 (−0.192, 0.062)	0.936 (0.884, 0.965)	0.281	0.780
Left	6.904 ± 1.059 (4.713–8.470)	6.951 ± 1.183 (4.637–8.890)	0.047 (−0.135, 0.229)	0.873 (0.777, 0.930)	0.400	1.109
Front-left	7.342 ± 1.148 (4.433–9.133)	7.428 ± 1.217 (4.367–9.000)	0.087 (−0.053, 0.227)	0.931 (0.875, 0.962)	0.312	0.864
**PST—Sway indices**						
AP	0.033 ± 0.015 (0.017–0.093)	0.032 ± 0.014 (0.017–0.093)	−0.001 (−0.003, 0.001)	0.915 (0.849, 0.954)	0.004	0.012
ML	0.021 ± 0.009 (0.010–0.050)	0.020 ± 0.008 (0.010–0.050)	−0.001 (−0.002, 0.001)	0.866 (0.764, 0.926)	0.003	0.008
OA	0.037 ± 0.010 (0.020–0.077)	0.038 ± 0.010 (0.020–0.077)	0.001 (−0.000, 0.003)	0.903 (0.828, 0.947)	0.003	0.008
**PST—Stability indices**						
AP	0.278 ± 0.119 (0.153–0.697)	0.290 ± 0.128 (0.157–0.727)	0.012 (−0.001, 0.025)	0.943 (0.897, 0.969)	0.029	0.082
ML	0.190 ± 0.076 (0.087–0.417)	0.189 ± 0.085 (0.070–0.420)	0.000 (−0.011, 0.011)	0.910 (0.838, 0.951)	0.024	0.067
OA	0.373 ± 0.153 (0.190–0.787)	0.399 ± 0.170 (0.193–0.843)	0.026 (0.011, 0.041)	0.946 (0.903, 0.971)	0.038	0.104

Values are mean ± SD (minimum–maximum) in the raw Al Balance output scale. Mean difference = Day 7 − Day 1, with confidence intervals from the paired-samples *t* distribution. ICC(A,1), two-way mixed-effects, absolute-agreement, single-measure intraclass correlation coefficient calculated from session-level scores, each defined as the mean of three trials. SEM = SD_pooled_ × √(1 − ICC), where SD_pooled_ = √[(SD_Day 1_^2^ + SD_Day 7_^2^)/2]; MDC_95_ = 1.96 × √2 × SEM. LoS, Limits of Stability; PST, Postural Stability Test; AP, anteroposterior; ML, mediolateral; OA, overall; CI, confidence interval; SD, standard deviation; SEM, standard error of measurement; MDC_95_, minimal detectable change at the 95% confidence level. All analyses are based on 40 paired observations. PST and LoS reliability estimates are interpreted as within-device measurement properties of Al Balance. Values are rounded to three decimals; all calculations used unrounded data.

## Data Availability

The data presented in this study are available on request from the corresponding author.

## Supplementary Materials

The following supporting information can be downloaded at: https://www.mdpi.com/article/10.3390/s26216757/s1. Table S1: Sphericity tests and Greenhouse–Geisser corrections for all repeated-measures models with more than two levels; Table S2: Non-parametric confirmation of the a priori sensory hypotheses: Wilcoxon signed-rank tests on participant-level contrasts; Table S3: Between-device Spearman correlations of participant-level sensory response magnitudes, with Holm adjustment.

## Abbreviations

The following abbreviations are used in this manuscript:

AI	Artificial intelligence
ANOVA	Analysis of variance
AP	Anteroposterior
BBS	Biodex Balance System SD
BCT	Bilateral Comparison Test
BMI	Body mass index
CI	Confidence interval
COSMIN	COnsensus-based Standards for the selection of health Measurement INstruments
EC	Eyes closed
EO	Eyes open
ICC	Intraclass correlation coefficient
IQR	Interquartile range
LoS	Limits of Stability
mCTSIB	Modified Clinical Test of Sensory Interaction on Balance
MDC_95_	Minimal detectable change at the 95% confidence level
ML	Mediolateral
OA	Overall
PST	Postural Stability Test
r_rm_	Repeated-measures correlation
SD	Standard deviation
SEM	Standard error of measurement
