# Supplementary material for: Construct Validity and Test–Retest Reliability of the Al Balance Stabilometric System in Healthy Young Adults
Source: Sensors (Basel). 2026 Oct 26;26(21):6757. doi: 10.3390/s26216757 (PMC13765734; doi:10.3390/s26216757)
Supplement: Supplementary file 1 [file sensors-26-06757-s001.zip › sensors-4574531-supplementary.pdf]

Supplementary Material

Supporting statistics for the primary mCTSIB construct-validity analyses and the within-device LoS analyses summarized in Tables S1–S3. These supplementary tables provide sphericity corrections, non-parametric confirmation of the a priori sensory hypotheses, and Holm-adjusted correlations of participant-level sensory response magnitudes.

Table S1. Sphericity tests and Greenhouse–Geisser corrections for all repeated-measures models with more than two levels.

Model	Effect	Mauchly W	p	F (df)	ε	Corrected df	p (GG)	ηp²
**mCTSIB, Overall**	Condition	0.484	< 0.001	164.20 (3, 117)	0.713	2.14, 83.40	< 0.001	0.808
	Device × Condition	0.484	< 0.001	20.85 (3, 117)	0.745	2.23, 87.13	< 0.001	0.348
**mCTSIB, AP**	Condition	0.546	< 0.001	164.91 (3, 117)	0.755	2.26, 88.33	< 0.001	0.809
	Device × Condition	0.546	< 0.001	20.80 (3, 117)	0.770	2.31, 90.12	< 0.001	0.348
**mCTSIB, ML**	Condition	0.692	0.016	110.86 (3, 117)	0.853	2.56, 99.75	< 0.001	0.740
	Device × Condition	0.692	0.016	10.47 (3, 117)	0.870	2.61, 101.81	< 0.001	0.212
**LoS, Al Balance**	Direction (within device)	0.005	< 0.001	76.02 (7, 273)	0.359	2.51, 97.88	< 0.001	0.661
**LoS, Biodex**	Direction (within device)	0.307	0.026	3.92 (7, 273)	0.778	5.45, 212.45	0.001	0.091

ε, Greenhouse–Geisser epsilon; GG, Greenhouse–Geisser corrected; ηp², partial eta squared; mCTSIB, Modified Clinical Test of Sensory Interaction on Balance; LoS, Limits of Stability. The mCTSIB rows summarize the four-level standardized condition and device × condition models used in the primary construct-validity analysis. The final LoS rows summarize the within-device direction effects reported in Table 2. The 2 × 2 vision × surface models in Table 1 and device × side models for BCT are not listed because two-level within-subject factors do not require a sphericity correction.

Table S2. Non-parametric confirmation of the a priori sensory hypotheses: Wilcoxon signed-rank tests on participant-level contrasts.

Device	Axis	Vision effect		Surface effect		Vision × Surface	
		p	Direction	p	Direction	p	Direction
**Al Balance**	Overall	< 0.001	92.5%	< 0.001	97.5%	< 0.001	80.0%
	AP	< 0.001	90.0%	< 0.001	92.5%	< 0.001	80.0%
	ML	< 0.001	87.5%	< 0.001	92.5%	< 0.001	82.5%
**Biodex**	Overall	< 0.001	100.0%	< 0.001	85.0%	< 0.001	77.5%
	AP	< 0.001	100.0%	< 0.001	72.5%	0.001	75.0%
	ML	< 0.001	97.5%	< 0.001	82.5%	< 0.001	82.5%

Contrasts were computed per participant as follows. Vision effect: mean of the two eyes-closed conditions minus mean of the two eyes-open conditions. Surface effect: mean of the two foam conditions minus mean of the two firm conditions. Vision × Surface: (eyes-closed foam − eyes-open foam) − (eyes-closed firm − eyes-open firm). Tests are one-sided against the a priori prediction that each contrast is positive, that is, that the manipulation increases sway. "Direction" gives the percentage of the 40 participants whose contrast moved in the hypothesized direction. These tests were performed because Shapiro–Wilk tests indicated departures from normality in 16 of the 24 device × axis × condition cells; all 18 contrasts remained significant, so the parametric conclusions in Table 1 are not artifacts of distributional violations. The smallest effect was the Biodex AP vision × surface contrast, $p = 0.001$.

Table S3. Between-device Spearman correlations of participant-level sensory response magnitudes, with Holm adjustment.

Axis	Response Magnitude	Al Balance (Mean ± SD)	Biodex (Mean ± SD)	ρ	p	p (Holm)
**Overall**	Vision effect	1.55 ± 1.16	1.08 ± 0.48	0.411	0.008	0.076
	Surface effect	2.11 ± 1.17	0.34 ± 0.35	−0.145	0.371	1.000
	Vision × Surface	1.71 ± 1.97	0.45 ± 0.67	−0.033	0.839	1.000
**AP**	Vision effect	1.43 ± 0.91	0.82 ± 0.33	0.304	0.056	0.394
	Surface effect	1.59 ± 1.13	0.19 ± 0.26	0.108	0.507	1.000
	Vision × Surface	1.50 ± 1.79	0.23 ± 0.43	−0.362	0.022	0.175
**ML**	Vision effect	0.84 ± 0.87	0.66 ± 0.38	0.093	0.570	1.000
	Surface effect	1.29 ± 0.83	0.27 ± 0.31	0.006	0.973	1.000
	Vision × Surface	1.26 ± 1.60	0.41 ± 0.56	0.135	0.406	1.000

Response magnitudes are defined as in Table S2 and are expressed in each system's own display unit, so the Al Balance and Biodex columns are not numerically comparable; the correlation is unaffected by scale. Positive means indicate deterioration in the hypothesized direction, which was observed for every contrast on both systems. The Holm adjustment was applied across all nine tests, comprising three response magnitudes in each of three axes. No correlation survived correction. Restricting the family to the three contrasts of the primary Overall axis would give an adjusted p of 0.025 for the vision effect. Difference scores compound the measurement error of the two conditions from which they are formed, so weak correlations are expected here and do not contradict the repeated-measures correlations reported in Table 3, which are estimated across all four conditions simultaneously.
